# Brainstem Encephalitis With Conflicting Autoimmune and Infectious Markers: A Case Report

**DOI:** 10.7759/cureus.107784

**Published:** 2026-04-27

**Authors:** Ismail Yuce, Mustafa E Tavsanli

**Affiliations:** 1 Operating Room Services Program, Vocational School of Health Services, Acibadem Mehmet Ali Aydinlar University, Istanbul, TUR; 2 Electroneurophysiology Program, Vocational School of Health Services, Acibadem Mehmet Ali Aydinlar University, Istanbul, TUR

**Keywords:** astroblastoma, case report, demyelinating autoimmune diseases, encephalitis, paraneoplastic syndromes

## Abstract

The differential diagnosis of inflammatory diseases of the central nervous system has a very wide spectrum, and the diseases may mimic one another. Although modern techniques help clinicians make a final diagnosis, there still can be challenges and pitfalls in some cases. We present an 18-year-old female patient who presented with signs of brainstem encephalitis, which was accompanied by a small hyperintense lesion in the frontal lobe. The radiological findings were nonspecific, and her cerebrospinal fluid findings were normal. However, she had positive centromere antibody in the serum and positive CD45 antibody in the minor salivary gland biopsy. These markers led to the initial diagnosis of a rheumatological disorder, vasculitis, and immunosuppressive treatment was started. However, in the following weeks, her condition got worse. Further investigations showed positivity on the interferon-g release assay test. This changed the diagnosis to tuberculosis, advancing under immunosuppressive treatment. Despite the given antituberculosis treatment, the lesion in the frontal lobe eventually progressed and became eligible for biopsy, while the lesion in the brainstem was stable. The biopsy showed the lesion in the frontal lobe as “astroblastoma.” The brainstem lesion started to recover after the removal of the tumor, and it was diagnosed as a paraneoplastic complication of the malignancy. This case showed how the differential diagnosis can be challenging and that the markers can sometimes be misleading. We would like to present the case for its educational value and its rarity.

## Introduction

Inflammatory diseases of the brain remain a major cause of disability in neurology [1]. The differential diagnosis of these diseases has a very wide spectrum, including demyelinating diseases, vasculitis, autoimmune encephalitis, neoplasia, etc., which may mimic each other and require careful evaluation [2]. The use of magnetic resonance imaging (MRI) and the availability of the markers in serum or cerebrospinal fluid (CSF) help clinicians to make a final diagnosis [3]. However, in some cases, even these advanced techniques are insufficient to determine the nature of the lesion, or there may be confusing results in the laboratory findings, which would be a great challenge. We present a young female patient with brainstem encephalitis accompanied by a separate frontal lesion, in whom radiological, immunological, and infectious findings created significant diagnostic uncertainty. This case required a multidisciplinary approach and highlights the challenges of distinguishing between inflammatory, infectious, and neoplastic processes in the central nervous system (CNS).

## Case presentation

**Figure 1:**
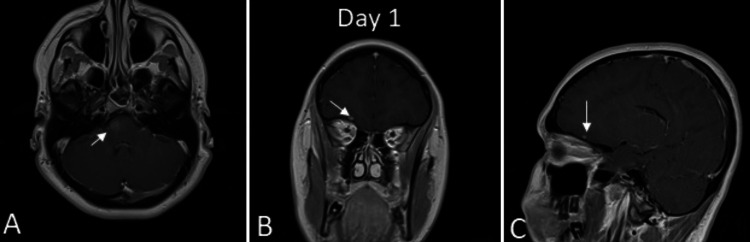
Initial MRIs of the patient (A) The anterior midline and anterior right side of the medulla oblongata-bulbus junction, which shows patchy gadolinium enhancement. (B,C) The basal area of the right frontal lobe, with a diameter of 5 mm, which shows patchy gadolinium enhancement MRI: magnetic resonance imaging

An 18-year-old female patient was admitted to our clinic in March 2024 with a complaint of double vision. Her complaint had progressed within two weeks, and a deviation of her right eye was eventually noticed by her parents. She also felt mild weakness in her left arm and leg during this period. Her examination showed left-sided hemiparesis at a level of -5/5, increased left deep tendon reflexes, and limited deviation of the right eye to the lateral side. She had been evaluated with a cranial MRI before her admission to our clinic, and the result showed hyperintensity in the anterior midline and anterior right side of the medulla oblongata-bulbus junction, with patchy gadolinium enhancement. There was another lesion in the basal area of the right frontal lobe, with a diameter of 5 mm, also enhancing with gadolinium. An investigation of a brainstem lesion with magnetic resonance (MR)-spectroscopy showed no significant neuronal markers, but only a high lipid-lactate peak (Figure 1). She was admitted with a prediagnosis of demyelinating disease. Although the MRI findings were not typical for multiple sclerosis, they were also not commented on as a neoplasia. The CSF study showed no cells, clear bacterial, viral, and fungal cultures, normal biochemical findings, a normal immunoglobulin G (IgG) index, and negative oligoclonal banding. Serum anti-neuromyelitis optica (Anti-NMO) and anti-myelin oligodendrocyte glycoprotein (anti-MOG) antibodies were negative. Serum and urine protein electrophoresis were normal. The polymerase chain reaction (PCR) study for tuberculosis was also negative. The initial diagnosis was a demyelinating disease, and she was treated with intravenous corticosteroid 1,000 mg/day for seven days.

The markers of systemic vasculitis were all negative, except that the serum extractable nuclear antigen panel showed a centromere CENP-B antibody of 4+, and the antinuclear antibody (ANA) level was positive in a centromeric pattern at a 1:2560 titration. She was consulted by rheumatology on May 2, 2024, for a control MRI, which showed findings similar to the first MRI. The positivity of the centromere CENP-B antibody was thought by the rheumatologist to be related to Sjögren’s disease or scleroderma. The patient did not report any systemic complaints that could be related to these pathologies, but the rheumatologist suggested a minor salivary gland biopsy, echocardiography, and an eye examination for uveitis. Echocardiography and eye examination showed normal findings; however, the minor salivary gland biopsy was positive for CD45 antibody. The steroid treatment was tapered with an intravenous 500 mg dose for three days, then switched to oral tablets at 1 mg/kg per day (initial dose 80 mg) and planned to be tapered by 8 mg/week. At the end of the treatment, the movements of the right eye started to recover, and the hemiparesis on the left side became slightly better.

Two weeks later, while she was still under 48 mg/day oral steroid treatment, her complaints progressed again. The left-sided hemiparesis became at a level of 4/5, and the right eye’s limited deviation to the lateral side also increased. Another control MRI was ordered, which showed increased contrast enhancement of the brainstem lesion, but no new lesions. The differential diagnosis of the MRI finding included vasculitis, demyelinating diseases, encephalitis, and, with a low probability, lymphoma and neoplasia. Rheumatology evaluated the patient again. Although she did not show a systemic presentation of a rheumatological disease, the positivity of the aforementioned antibodies, the MRI pattern, and the progression of the complaints led them to accept the condition as a probable vasculitis. Corticosteroid 500 mg intravenously for three days, followed by 32 mg orally, was started by the rheumatologist in combination with mycophenolate mofetil 2 x 1 g.

Ten days later, she showed no significant recovery. The rheumatology department discussed the patient again and noted that the condition was unlikely to be a rheumatological disease; therefore, neoplastic conditions should be excluded. However, the lesion in the brainstem was not available for biopsy due to a high risk of damage, and the lesion in the frontal lobe was too small and again not suitable for biopsy. Mycophenolate mofetil was stopped, and a control CSF analysis was performed. The findings of the CSF study were clear, and there was no malignant cell in the CSF. Thoracic, abdominal, and pelvic computed tomography studies, breast ultrasonography, and gynecological evaluation did not show evidence of malignancy. The positron emission tomography (PET) study showed nonspecific enhancement in the brainstem but no evidence of a neoplastic focus. Paraneoplastic antibody scan and antibodies for autoimmune encephalitis were all negative. The human leukocyte antigen gene for Behçet’s disease was also negative. The T-cell interferon-gamma release assay (IGRA) test (QuantiFERON® TB-Gold) was positive (0.60 IU/mL), and the suspicion for tuberculosis increased. We performed bronchoscopy to take a sample to search for tuberculosis and malignancies in the respiratory fluids. However, the cytological evaluation of respiratory system secretions was clear. The infectious diseases specialist recommended anti-TB treatment (isoniazid 1 x 300 mg, rifampin 1 x 600 mg, ethambutol 1 x 1,500 mg, pyrazinamide 1 x 2,000 mg) based on a positive IGRA test and an unidentified brain lesion that showed progression, probably due to immunosuppressive treatments. The lesion in the frontal lobe was also thought to be compatible with tuberculoma by the radiologist, in light of these new results. We started the recommended treatments and stopped the steroid treatment. We thought that the brainstem involvement could be an autoimmune reaction to tuberculosis infection and started intravenous immunoglobulin treatment at 0.4 g/kg/day for five days.

**Figure 2:**
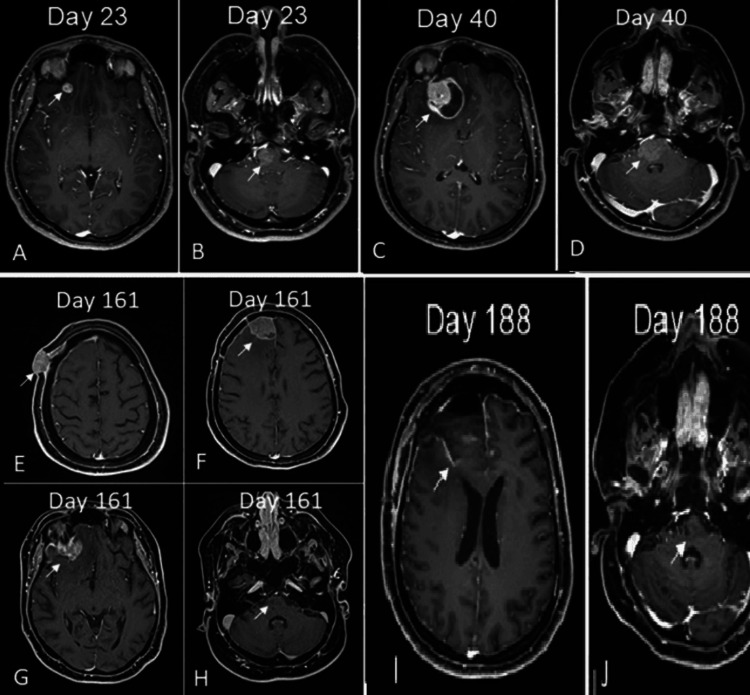
Follow-up MRIs of the patient (A,B) The lesion at the anterior midline and anterior right side of the medulla oblongata-bulbus junction and the basal area of the right frontal lobe, showing patchy gadolinium enhancement. (C,D) One month later, the lesion in the frontal lobe was growing in an expansile pattern. (E-G) The recurrent lesions at the basal area of the right frontal lobe and invasion to the skin area, which show patchy gadolinium enhancement. (H) The lesion at the anterior midline and anterior right side of the medulla oblongata-bulbus junction, which shows a significant decrease in gadolinium enhancement. (I) The postoperative images of the recurrent lesion at the basal area of the right frontal lobe. (J) The lesion at the anterior midline and anterior right side of the medulla oblongata-bulbus junction shows a significant decrease in gadolinium enhancement MRI: magnetic resonance imaging

Within one week, she had resistant headaches, a swallowing problem, and dysarthria. Her gag reflex was decreased. Left-sided hemiparesis progressed to 2/5 in the proximal muscles and to 3/5 in the distal muscles. Control MRI showed increased edema in the frontal lesion, but the brainstem lesion was relatively stable. This finding supported our concern of two lesions were two different pathologies. Neurosurgery recommended an excisional biopsy of the frontal lesion, since it was large enough to be operated on at that point (Figures 2A-2D).

The operation took place on May 21, 2024. The biopsy showed a well-demarcated lesion, growing in an expansile pattern with moderate-to-high cellular activity and a papillary configuration. The cells showed large cytoplasm and high mitotic activity, with necrosis. The lesion was diagnosed as astroblastoma according to the World Health Organization (WHO) 2021 classification, and it could not be graded.

**Figure 3:**
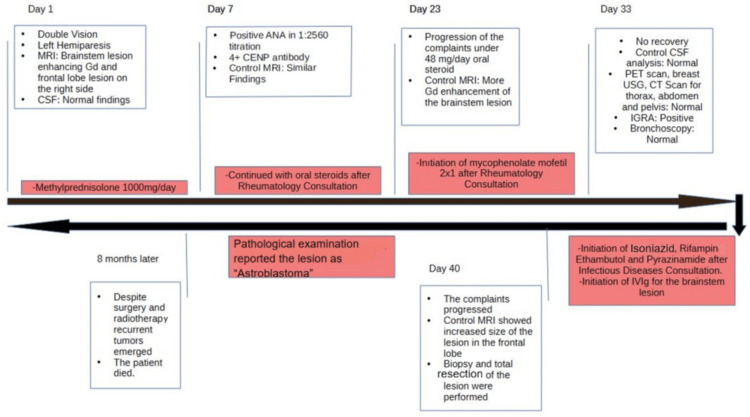
Timeline of the case

The total resection of the lesion in the frontal lobe was performed. After the surgery, she had local radiotherapy to the resection area and stereotaxic radiotherapy to the brainstem as well, since the nature of the lesion in the brainstem was still not clear. After the operation, the MRIs revealed that the gadolinium enhancement of the brainstem had dramatically recovered. However, she had recurrent lesions in the right frontal area. She was operated on once more, and despite surgery and radiotherapy, she passed away at the end of 2024 (Figures 2E-2J). Table 1 summarizes the blood and CSF study results of the patient. Figure 3 summarizes the timeline of her follow-up.

**Table 1:** Summary of the blood and CSF studies of the patient CSF: cerebrospinal fluid; PCR: polymerase chain reaction; IGRA: interferon-gamma release assay; c-ANCA: cytoplasmic anti-neutrophil cytoplasmic antibodies; p-ANCA: perinuclear anti-neutrophil cytoplasmic antibodies; anti-ds-DNA: anti-double-stranded DNA antibodies; COI: cutoff index; VDRL: Venereal Disease Research Laboratory; RPR: rapid plasma reagin; anti-NMO: anti-neuromyelitis optica; anti-MOG: anti-myelin oligodendrocyte glycoprotein

Study name	Result (March 2024)	Result (May 2024)
CSF cell count	No cells	No cells
CSF glucose	60 mg/dL	74 mg/dL
CSF protein	22.5 mg/dL	26.4
CSF sodium	147 mmol/L	145 mmol/L
CSF potassium	2.86 mmol/L	2.79 mmol/L
CSF chlorine	125 mmol/L	123 mmol/L
CSF/serum IgG ratio	1.53	1.8
IgG index	0.44	0.42
CSF oligoclonal banding	Pattern type 1	Pattern type 1
CSF angiotensin-converting enzyme	Negative	-
CSF culture (bacterial and fungal)/viral PCR study	Sterile/negative	Sterile/negative
CSF tuberculosis DNA	Negative	Negative
CSF acid-resistant bacteria	-	Negative
CSF culture for Mycobacteria	-	Negative
CSF *Toxoplasma gondii* DNA	-	Negative
Bronchoalveolar lavage acid-resistant bacteria	-	Negative
Bronchoalveolar lavage tuberculosis DNA	-	Negative
IGRA (QuantiFERON® TB-Gold)	-	Positive 0.60 IU/mL
Serum c-ANCA, p-ANCA, anti-ds-DNA, anticardiolipine IgM/IgG antibodies, mitochondrial antibody, antithrombin-III, lupus anticoagulant, protein C, and S activities	Negative/in normal limits	-
Serum antiganglioside profile (GM-1, GM-2, GM-3, GD1a, GD1b, GQ1b, GT1b)	Negative IgG and IgM antibodies	-
Serum HIV antibody + p24 antigen	Negative, 0.23 COI	Negative, 0.164 COI
Serum VDRL/RPR	Negative	-
Serum *Borrelia burgdorferi* IgG antibody	Negative	Negative
Serum anti-MOG/anti-NMO	Negative/negative	-
Serum ENA profile (anti-SsA, SsB, Sm, RNP/Sm, ScL70, J0-1, Centromere (CENP-B), Histone, Nucleosome, Ro-52, ribosomal P protein, Pm/Scl, Mi-2, Ku antibodies)	CENP-B antibody 4+	-
Serum antinuclear antibody	Positive, centromeric, 1:2560 titration	-
Serum autoimmune encephalitis panel (GAD65, NMDAR, GABAAR, GABABR, IgLON5, AMPAR2, DPPX, LGI1, CASPR2, mGLUR5, m GLUR1, glycine receptor antibody)	-	Negative
Serum paraneoplastic antibody panel (anti-amphiphysin, anti-CV2, anti-PNMA2, anti-Ri, anti-Yo, anti-Hu, anti-recoverin, anti-SOX1, anti-Titin, anti-Zic4, anti-GAD65, anti-Tr)	-	Negative

## Discussion

This case illustrates a complex diagnostic process that requires a systematic evaluation of multiple potential etiologies. To clarify the clinical reasoning, the differential diagnosis can be structured into several major categories: demyelinating diseases, autoimmune/rheumatological disorders, infectious etiologies, and neoplasies.

She had an MRI, which revealed two separate foci at the beginning, with two distinct features. Demyelinating disorders, including multiple sclerosis and neuromyelitis optica spectrum disorders, were initially considered due to the brainstem involvement and clinical presentation. However, the lesions did not fulfill the defined criteria of multiple sclerosis [4]. Furthermore, there was no oligoclonal band positivity, no increased IgG index, and no positivity of anti-MOG and anti-NMO antibodies.

Lesions in autoimmune and rheumatological diseases may also show similar patterns on MRI, and differentiation may be impossible even with multiple techniques, ultimately leading to misdiagnosis [5]. CSF and serum studies in the presented case did not support intrathecal inflammation or infection. We accepted the case as a noninfectious inflammatory disease based on the results. In our case, the presence of centromere (CENP-B) antibodies and ANA positivity initially raised suspicion for an autoimmune disorder such as systemic sclerosis or Sjögren’s disease. However, these antibodies are not disease-specific and may be detected in a variety of conditions. CENP-B antibody is positive in 20%-40% of patients with systemic sclerosis, with specificity >95%, but it can also be present in systemic lupus erythematosus, rheumatoid arthritis, Sjögren’s disease, and malignancies [6,7]. Therefore, these findings should be interpreted with caution, and the possibility of an incidental positivity must be kept in mind.

Despite the patient having no complaints related to a rheumatological disorder, the rheumatologist recommended further evaluation, and a biopsy of the minor salivary glands was performed. The biopsy showed CD45+ cells, which could be consistent with Sjögren’s disease but is not specific, as it may reflect nonspecific lymphocytic infiltration. Although she had no other signs of the disease, in combination with the MRI and the antibody test results, the treatment was adjusted for a vasculitic process (i.e., mycophenolate mofetil and steroids) by the rheumatologist. However, her complaints progressed under this treatment. The absence of systemic involvement and a poor response to immunosuppressive treatment led us to exclude a rheumatological process, and the rheumatologists advised searching for an underlying malignancy, since the aforementioned markers, especially the CENP-B antibody, can be associated with malignancies as well as rheumatological diseases [8,9].

At this point, we stopped the treatment and widened our search for a possible neoplasia. However, no proof of a tumor was found in the radiological scans, the PET scan, or the blood sample. Although the PET scan showed increased enhancement in the brainstem, it was commented on as a nonspecific finding of an inflammatory process. The control CSF was still clear.

The progression of symptoms under immunosuppressive therapy led us to consider a relapse of an infection, and we repeated tests for infectious diseases. The result of the IGRA test was found positive. This finding led us to consider tuberculosis, particularly as an etiologic factor. The frontal lesion was said to be compatible with a tuberculoma by the radiologist (the MRI images of the frontal lesion were commented as liquefaction, compatible with caseation necrosis). Although these comments led us to consider the lesion in the frontal lobe to be a tuberculoma, it must be kept in mind that IGRA positivity cannot distinguish between active and latent infection and may also yield false-positive results [10-12].

Furthermore, this case lacked microbiological evidence of tuberculosis (i.e., negative PCR, cultures, and bronchoscopy findings), which significantly limited the diagnostic certainty of active tuberculosis. Even though there was a strong limitation for the diagnosis of an active tuberculosis infection and IGRA positivity alone was insufficient to establish this diagnosis, antituberculosis treatment was started due to the progression of her complaints after the immunosuppressive treatment. The brainstem lesion was thought to be an autoimmune reaction to the frontal lesion, which is thought to be a tuberculoma. These decisions were made under diagnostic uncertainty, and they were another pitfall in this case.

After the initiation of the antituberculosis treatments, the patient had drug-resistant headaches, and her neurological condition continued to decline. The control MRI showed an increase in the size of the frontal lesion and showed edema around it. However, the lesion in the brainstem was relatively stable. This finding was also thought to be supportive of an activation of tuberculosis due to the use of immunosuppressant treatments. Although neoplastic etiologies were initially considered less likely due to atypical imaging findings and nonsupportive MR-spectroscopy results, the progression of the disease despite the medical treatments and the eventual growth of the frontal lesion led us to perform a biopsy.

The result of the biopsy showed that the lesion was astroblastoma, with a high level of mitotic activity. Astroblastoma is a rare tumor of the central nervous system and has challenges both in diagnosis and definition [13]. There is still an ongoing debate about the classification and histogenesis of astroblastomas, and we could not grade the tumor according to the WHO guidelines, which were published in 2021 [13,14].

In our case, we initially focused on the brainstem lesion, which was responsible for the neurological symptoms. The radiological features, including the MR-spectroscopy features and the progression pattern of this lesion, were not similar to the frontal lesion. Furthermore, the MR-spectroscopy findings were not supportive of a neoplastic process. In the literature, there are defined MRI patterns of paraneoplastic syndrome, which also include a pattern of brainstem encephalitis [15]. This MRI pattern mimics other inflammatory lesions, such as Behçet’s disease, systemic lupus erythematosus, Bickerstaff encephalitis, Miller Fischer syndrome [16,17]. Demyelinating diseases, such as multiple sclerosis, neuromyelitis optica spectrum disorders, and myelin oligodendrocyte glycoprotein antibody-associated disease, should also be differentiated [15]. After the surgery, the control MRI showed that the brainstem lesion was not enhancing with contrast. This raises the possibility of a paraneoplastic inflammatory process. However, in the absence of paraneoplastic antibodies and histopathological confirmation, this interpretation should be considered as probable.

Emerging experimental and clinical studies have highlighted the role of immune-mediated and interferon-associated pathways in central nervous system inflammatory disorders. In particular, interleukin-12-related signaling and LINE-1 retrotransposon activation with downstream type I interferon responses have been implicated in preclinical models of neuroinflammation and demyelination. In addition, clinical neuroimmunological studies have suggested that interferon regulatory pathways and biomarkers such as BTK and YKL-40 may contribute to disease activity and heterogeneity in autoimmune CNS disorders. These findings collectively suggest that molecular and immunological markers may, in the future, provide additional support in the differential diagnosis of complex neurological syndromes [18-20]. To our knowledge, there has been no defined case showing similarity to our case.

## Conclusions

In this case, the brainstem lesion was most likely a paraneoplastic inflammatory process associated with astroblastoma; however, this interpretation cannot be confirmed due to the lack of histopathological and serological evidence.

The presence of autoimmune markers such as CENP-B and CD45 antibodies was a pitfall in our case, as these findings are not disease-specific and may be related to malignancy or incidental immune activation rather than rheumatological disease. Similarly, IGRA positivity introduced further diagnostic uncertainty, as it cannot distinguish between acute and latent mycobacterial infection.

This case highlights the importance of a structured differential diagnosis approach and emphasizes that atypical or treatment-resistant central nervous system inflammatory presentations should prompt thorough evaluation for an underlying malignancy.
